# Chemotherapy-induced release of circulating-tumor cells into the bloodstream in collective migration units with cancer-associated fibroblasts in metastatic cancer patients

**DOI:** 10.1186/s12885-020-07376-1

**Published:** 2020-09-11

**Authors:** Nerymar Ortiz-Otero, Jocelyn R. Marshall, Bradley Lash, Michael R. King

**Affiliations:** 1 Meinig School of Biomedical Engineering, Cornell University, Ithaca, NY 14850 USA; 2 Guthrie Clinical Research Center, Sayre, PA 18840 USA; 3 Department of Biomedical Engineering, Vanderbilt University, Nashville, TN 37235 USA

**Keywords:** Circulating tumor cells, Cancer-associated fibroblast, Cancer prognosis, Chemotherapy, TRAIL-based liposomal therapy

## Abstract

**Background:**

Recent studies have shown that chemotherapy destabilizes the blood vasculature and increases circulating tumor cell (CTC) influx into the circulation of metastatic cancer patients (Met-pa). CTCs are a precursor of cancer metastasis, in which they can migrate as single CTCs or as CTC clusters with stromal cells such as cancer-associated fibroblasts (CAFs) as cell aggregates.

**Methods:**

Blood samples were collected from 52 Met-pa, and the number of CTC and CAF was determined along with the temporal fluctuation of these through the chemotherapy treatment.

**Results:**

In this study, CTC level was found to increase two-fold from the initial level after 1 cycle of chemotherapy and returned to baseline after 2 cycles of chemotherapy. Importantly, we determined for the first time that circulating CAF levels correlate with worse prognosis and a lower probability of survival in Met-pa. Based on the CTC release induced by chemotherapy, we evaluated the efficacy of our previously developed cancer immunotherapy to eradicate CTCs from Met-pa blood using an ex vivo approach and demonstrate this could kill over 60% of CTCs.

**Conclusion:**

Collectively, we found that CAF levels in Met-pa serve as a predictive biomarker for cancer prognosis. Additionally, we demonstrate the efficacy of our therapy to kill primary CTCs for a range of cancer types, supporting its potential use as an anti-metastasis therapy in the clinical setting.

## Background

Metastasis is the main cause of cancer-related mortality and morbidity [1]. When patients are diagnosed with metastatic cancer, the 5-year survival probability is less than 30% across many different cancer types [2]. During cancer metastasis, invasive tumor cells: (i) detach from the primary tumor, (ii) invade into the tumor stroma, (iii) intravasate into the blood vasculature, (iv) circulate in the bloodstream, becoming “circulating tumor cells” (CTCs), (v) extravasate into a distant tissue, and (vi) grow to develop into clinically detectable macrometastases [3]. Previous studies have established that CTCs migrate along with stromal cells, such as cancer-associated fibroblasts (CAF), as cell aggregates which can facilitate the survival and colonization in distant organs by tumor cells [4–6]. CAFs are the most abundant component of the tumor microenvironment, and are differentiated from normal fibroblasts by enhanced extracellular matrix protein production and the secretion of tumor-stimulating factor [7, 8]. Previous studies have established the pro-metastatic role of CAFs in primary and secondary locations through promoting tumor cell proliferation, invasion and enhancing colonization of disseminated cells in distant organs [4, 9, 10]. Recently, studies have found CAFs in blood samples from metastatic and localized cancer patients that correlate with disease progression in lung, breast and prostate cancer [11–13]. However, further studies are needed to fully validate and understand the correlation between CAF levels and prognosis in cancer patients.

Once CTCs develop into micrometastases, there is no treatment able to specifically eradicate cancer metastases. Currently, the first-line treatment used to prolong life expectancy and reduce metastatic tumors in Met-pa is systemic chemotherapy [14]. However, several studies have demonstrated that these treatments promote cancer metastasis by inducing blood and lymphatic vascular permeability, thereby enhancing the escape of tumor cells into the circulation [15, 16]. Several studies have demonstrated an increase in CTC levels post-treatment with several chemotherapeutic agents, including taxane-based drugs [15, 16]. Therefore, an adjuvant therapy that targets and kills CTCs in the circulation would be expected to enhance the efficacy of chemotherapeutic treatments when treating metastatic cancer in the clinical setting. In previous studies, we developed a cancer immunotherapy that consists of a CTC-targeted TNF-related apoptosis inducing ligand (TRAIL)-based liposomal therapy that targets and kills over 90% of CTCs in in vitro experiments and ~ 80% of CTCs in vivo using orthotopic murine models for prostate and breast cancer [17–19]. This TRAIL delivery approach consists of liposomes decorated with E-selectin and TRAIL on the liposomal surface. E-selectin is a natural adhesion protein displayed on activated endothelium, whereas TRAIL is a ligand that induces apoptosis in cancer cells via engagement with death receptors (DR) 4 and 5. Both of these death receptors are overexpressed on tumor cells but not normal cells [20]. The liposomal formulation attaches TRAIL to leukocytes via E-selectin adhesion under shear conditions. Due to leukocyte-tumor cell interactions, the therapy can dramatically reduce CTC levels by inducing apoptosis in these cells [16]. Here, we propose the use of TRAIL liposomes as an adjuvant therapy in combination with chemotherapy to enhance its efficacy in treating metastatic cancer.

In the present study, we quantified CTCs and CAFs released into the circulation in patients diagnosed with a range of metastatic cancer types, including colorectal, renal, breast, prostate, lung, endometrial, cervical, pancreatic, gastric and esophageal carcinoma. We found the presence of CAFs, and a positive correlation with cancer progression and unfavorable prognosis in metastatic cancers that have not been previously reported, such as renal, lung, endometrial, cervical, pancreatic, esophageal and gastric cancer. For the first time, we demonstrate the efficacy of our TRAIL-based liposomal therapy to kill primary CTCs in the flowing blood from Met-pa, which supports its potential use to eliminate CTCs from the circulation in combination with chemotherapy treatment to help reduce the metastatic burden in cancer patients and prolong life.

## Methods

### Blood sample collection from Met-pa

**Fig. 1:**
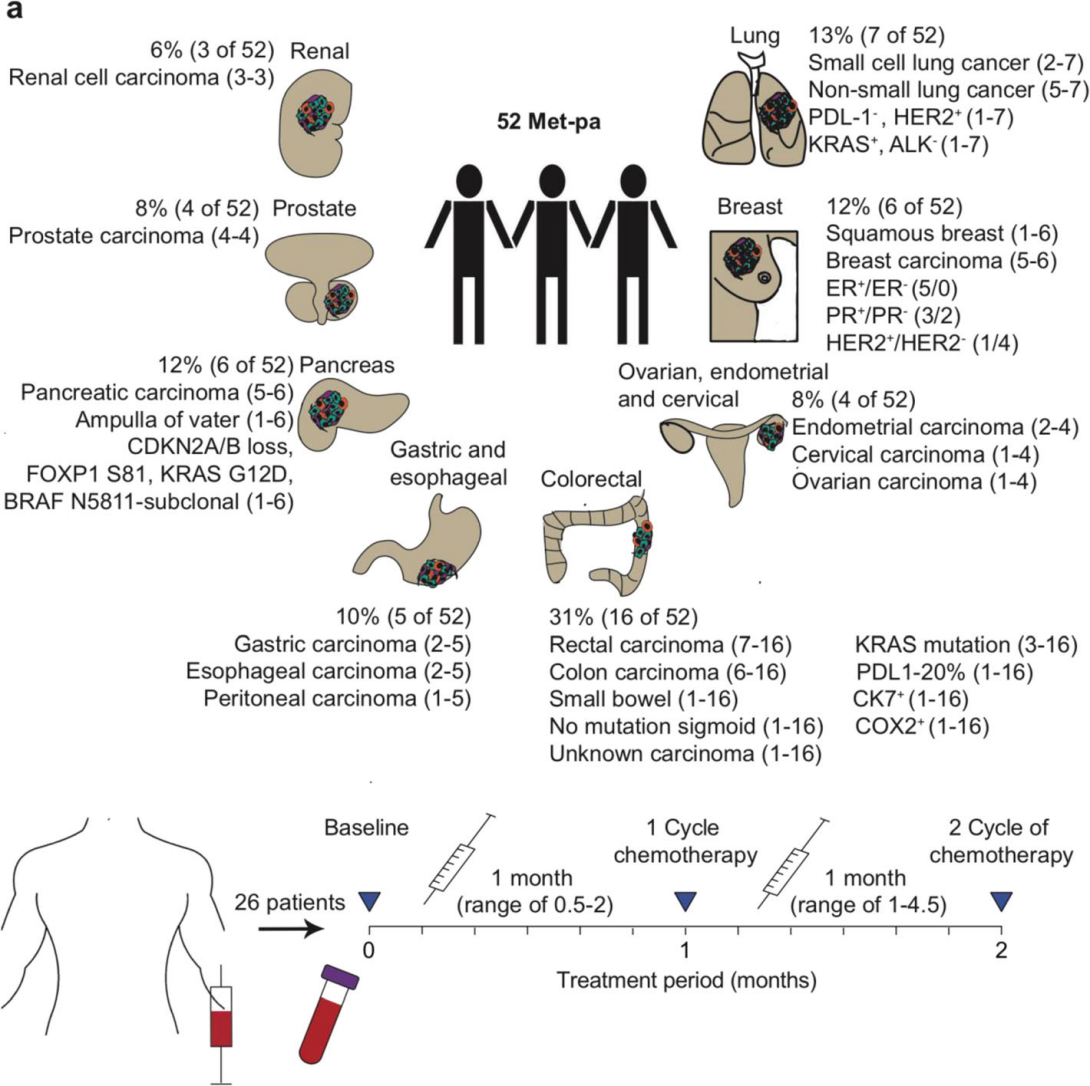
Diagram that display the blood collection from Met-pa and their clinical background information. **a** Blood samples were collected from a total of 52 patients diagnosed with a spectrum of cancer types at metastatic stage, including: renal, prostate, pancreatic, gastric, esophageal, colorectal, ovarian, endometrial, cervical, breast and lung carcinoma. From these 52 patients, 26 were followed through chemotherapy treatment. This figure was created by the authors for this article

Peripheral whole blood samples of 7.5 mL were collected from 52 Met-pa and 3 healthy volunteers after informed consent (Guthrie Clinic IRB#1808–45). All of the experiments were done in accordance with the U.S Federal Policy for the Protection of Human Subjects and approved by the Institutional Review Board of the Guthrie Clinic. The participants in this study were fully informed regarding the objective of the current study and written consent was obtained. Of these Met-pa, 48 and 44 samples were used to investigate the correlation between the initial CTC and CAF levels with cancer prognosis. Furthermore, 30 patient samples were collected through respective chemotherapy regimens at the following time points: (i) Baseline (pre-chemotherapy treatment), (ii) Following 1 cycle of chemotherapy and (iii) Following 2 cycles of chemotherapy. Finally, 12 patients were used as negative controls to fully determine the efficacy of our TRAIL-liposomal therapy. De-identified blood samples were shipped from the Guthrie Clinic to Vanderbilt University and processed within 24 h. Figure 1 and Table 1 show information related to the patient group that participated in this study. During blood collection, patients received the same chemotherapy regimen established at the baseline time point. In addition, the disease progression was determined by computerized tomography (CT scan) at each time point, where the patients were grouped as stable disease, disease progression and deceased. Stable disease is defined as when the metastatic burden has not changed. However, disease progression refers to patients displaying a growth of an existing metastatic lesion or the appearance of a new lesion in a distant organ. Patients that did not survive the cancer treatment were grouped as deceased (1–9 months of survival). After 1 year of blood collection, the clinical status of the patients analyzed in this study was determined and recorded. The patients were classified into three different categories: stable disease, progression and deceased patients. As stated above, stable disease and progression was determined using CT scan. However, when patients died, the date was recorded to determine the overall survival of this cohort of patients. To determine the effect of chemotherapy on CTC and CAF counts, patients that showed stable disease throughout chemotherapy treatment were considered for this part of the study. In this part, we wanted to investigate the CTC/CAF mobilization by treatment rather than by natural cancer progression.

**Table 1:** Clinical background information of the Met-pa participating in this study

Patient characteristics	
Total, *N*	52
Age (years), mean (range)	64 (40–83)
Gender, N (%)	
Female	26 (50%)
Male	26 (50%)
Type of cancer, N (%)	
Colon and rectum	16 (31%)
Breast	6 (12%)
Lung	7 (13%)
Pancreatic	6 (12%)
Gastric	3 (6%)
Esophageal	2 (4%)
Cervical, endometrial, ovarian	4 (8%)
Renal	3 (6%)
Prostate	4 (8%)
Metastasis location, N (%)	
Lung	16 (21%)
Bone	14 (18%)
Abdomen	5 (7%)
Liver	23 (30%)
Pancreas	2 (3%)
Brain	4 (5%)
Lymph nodes	3 (4%)
Pericardial effusion	2 (3%)
Omentum	2 (3%)
Cervical/uterus	4 (5%)
Scrotum	1 (1%)
Chemotherapy, N (%)	
5-Fluorouracil, Irinotecan, Oxaliplatin	10 (19%)
Taxanes (Taxol, Paclitaxel, Docetaxel)	5 (10%)
Alkylating agents (Carboplatin, Irinotecan, Cisplatin, Etopisode)	4 (8%)
Antimetabolites (Trabectedin, Capecitabine, Trifluridine, Pemetrexed)	6 (12%)
Inhibitor (Copanisilib, Pembrolizumab, Topotecan, Arbiraterone)	27 (52%)

### CTC and CAF isolation from Met-pa

Each blood sample was divided into 4 aliquots: (i) Untreated CTC identification, (ii) Untreated CAF identification, (iii) Treated with vehicle control and (iv) Treated with TRAIL therapy. To begin the CTC and CAF isolation process, the blood was placed over twice its volume of Ficoll (GE Healthcare) to separate the mononuclear cell layer, termed the buffy coat. Using a negative selection kit with CD45 magnetic beads (Mylteni Biotech), the CTCs were enriched following the manufacturer’s protocol [21]. In contrast, CAFs were isolated using a positive selection kit with anti-fibroblast magnetic beads (Mylteni Biotech) using the manufacturer’s protocol [22]. After isolation, the cells were fixed and cytospun onto glass microscope slides.

### Ex-vivo treatment of blood samples from Met-pa

A cone-and-plate viscometer was used to apply shear stress to the blood samples as an ex-vivo CTC microenvironment. This experimental setting has been extensively used by our research group to better characterize the efficacy of CTC-targeted therapeutic agents [17, 19, 23, 24]. An estimated 1–2 mL of blood aliquots were treated with 40 μL (~ 290 μg/mL of TRAIL) of vehicle control and TRAIL-liposomal solution and then placed in a cone-and-plate viscometer (Brookfield LVDVII) and sheared for 4 h. The cone-and-plate viscometers were incubated with 2 mL of 5% BSA for 30 min to block non-specific interactions with the surface. After 4 h, the blood was removed from the viscometer cup and spindle using 4 mL of HBSS buffer. CTCs were isolated from the sheared samples, as described above, and placed into cell culture overnight. After 1 day, the cells were recovered and stained with 100 μL of propidium iodide (BD) for 15 min. Cells were then fixed and cytospun onto glass microscope slides.

### Immunostaining of CTC and CAF from Met-pa

To identify and enumerate CTCs and CAFs from Met-pa, immunostaining of the isolated cells was carried out based on well-established biomarkers [21, 22]. Cells were hydrated and permeabilized using 100 μL of DPBS buffer (Gibco) for 25 min and 100 μL of 0.25% Triton-X (Sigma) for 15 min, respectively. Cells were then incubated with 100 μL of 5% BSA (Sigma) and 5% goat serum (Thermo Fisher) for 1 h to block nonspecific interactions. After blocking, cells were stained with 100 μL of 10 μg/mL of anti-CD45 conjugated with biotin (Clone HI30, Biolegend) for 45 min. The cells were then incubated with 100 μL of 10 μg/mL of Streptavidin-Alexa Fluor 594 (Biolegend) and 10 μg/mL of anti-cytokeratin conjugated with FITC (CK, Clone CAM5.2, BD) in 0.02% Tween-20 (Research Products) for 45 min [25]. To identify CAFs, anti-cytokeratin was replaced by anti-α-smooth muscle actin conjugated with eFluor 660 (α-SMA, Clone 1A4, eBioscience) [25]. After staining, the cells were washed with 200 μL of 0.02% Tween-20 three times. To determine the presence of CTC-CAF clusters, we stained for CD45, CK and α-SMA in the same sample. Finally, the cells were immersed with 15 μL of DAPI mounting media (Vectashield), covered with a coverslip and sealed with nail polish. Fluorescence micrographs were acquired using an LSM 710 META Inverted confocal microscope and analyzed using Image J software. The samples were imaged using 20x magnification and 5 pictures were taken per sample at random locations. The cell number was then scaled up using the frame area divided by the slide area. Tumor cells were enumerated using the following criteria: (i) Negative for CD45, (ii) Positive for cytokeratin and (iii) Intact nuclear staining with DAPI. CAFs were enumerated by using the following criteria: (i) Negative for CD45, (ii) Positive for α-SMA and (iii) Intact nuclear staining via DAPI.

### EMT phenotype in isolated CTCs

To evaluate EMT phenotype in CTCs, samples were stained using the protocol described in the previous section but targeting different biomarkers. At the time of antibody addition, cells were stained with 100 μL of 10 μg/mL anti-CD45 conjugated with biotin (Clone HI30, Biolegend) for 45 min. Then, cells were incubated with 100 μL of 10 μg/mL Streptavidin-Alexa Fluor 594 (Biolegend), 10 μg/mL of anti-cytokeratin conjugated with FITC (CK, Clone CAM5.2, BD) and 10 μg/mL of anti-vimentin conjugated with Alexa Fluor 647 (Clone W16220A, Biolegend) in 0.02% Tween-20 (Research Products) for 45 min. Fluorescence micrographs were acquired using a LSM 710 META inverted confocal microscope and analyzed using Image J software. To determine the EM phenotype, we used the cell lines MDA-MB-231 and MCF-7 to set the appropriate exposure time for these biomarkers. These two cell lines were used as a positive and negative control due to the strong expression of EMT independently. The E phenotype in CTCs was determined by using the following criteria: (1) positive for cytokeratin, (2) negative for vimentin, (3) negative for CD45 and (4) positive for DAPI (nuclear staining). However, the M phenotype in CTCs was determined by using the following criteria: (1) negative for cytokeratin, (2) positive for vimentin, (3) negative for CD45 and (4) positive for DAPI (nuclear staining). The samples were imaged using 20x magnification and 5 pictures were taken per sample at random locations. The cell number was then scaled up by the frame area divided by the slide area. The number of CTCs expressing either epithelial or mesenchymal markers were enumerated using confocal pictures and the percentages were calculated.

### Preparation of nanoscale liposomes

Multilamellar liposomes were prepared using a thin film method by combining the following lipids: Egg L-α-lysophosphatidylcholine (Egg PC, Avanti), egg sphingomyelin (Egg SM, Avanti), ovine wool cholesterol (Chol, Avanti) and 1,2-dioleoyl-sn-glyc- ero-3-[(N-(5-amino-1-carboxypentyl) iminodiacetic acid) succinyl] (nickel salt, Avanti) (DOGS NTA-Ni), using a weight ratio of 50%:30%:10%:10% (Egg PC: Egg SM: Chol: DOGS NTA-Ni). The lipids were mixed in a glass tube and placed in a vacuum chamber overnight to remove the organic solvent. The lipid pellet was hydrated using 700 μL of liposome buffer (20 mM HEPES, 150 mM NaCl, pH 7.5). Multilamellar liposomes were generated via 10 cycles of freezing (2 min) and thawing (3 min). To generate 100 nm unilamellar liposomes, the multilamellar liposomes were subjected to 10 extrusion cycles using polycarbonate membranes of two different sizes (200 nm and 100 nm) at 55 °C. Freshly made liposomes were incubated with E-selectin (17.5 μg/mL) and TRAIL (15 μg/mL) at 37 °C for 15 min. Functionalized liposomes were placed in a rotator at 4 °C prior to use. This liposome formulation has been previously used and characterized in Mitchell et al. 2014 [17].

### Statistical analysis

Patient blood samples at different time points through-out cancer treatment were considered biologically dependent repeats in the analysis of CTCs, CAFs and viable CTC percentage in this study. A normality test was performed before proceeding with the statistical analysis. For normally distributed data, paired *t-*test and one-way ANOVA test (repeated measures) were used to compare two or more groups, respectively. For non-normally distributed data, a Wilcoxon signed-rank test was used to compared two groups and a Friedman test was used to analyze more than two groups. For the survival curve, the Log-rank (Mantel-Cox) test was used. All of the tests were two-sided and performed at a significance level of α=0.05. A brief description is included in each figure legend indicating the following: number of samples, number of biological repeats, statistical test used and the *P-*value. The statistical analyses were performed using PRISM 6.0 software for Mac OS X.

## Results

### Elevated CAF numbers correlate with poor cancer prognosis in Met-pa

**Fig. 2:**
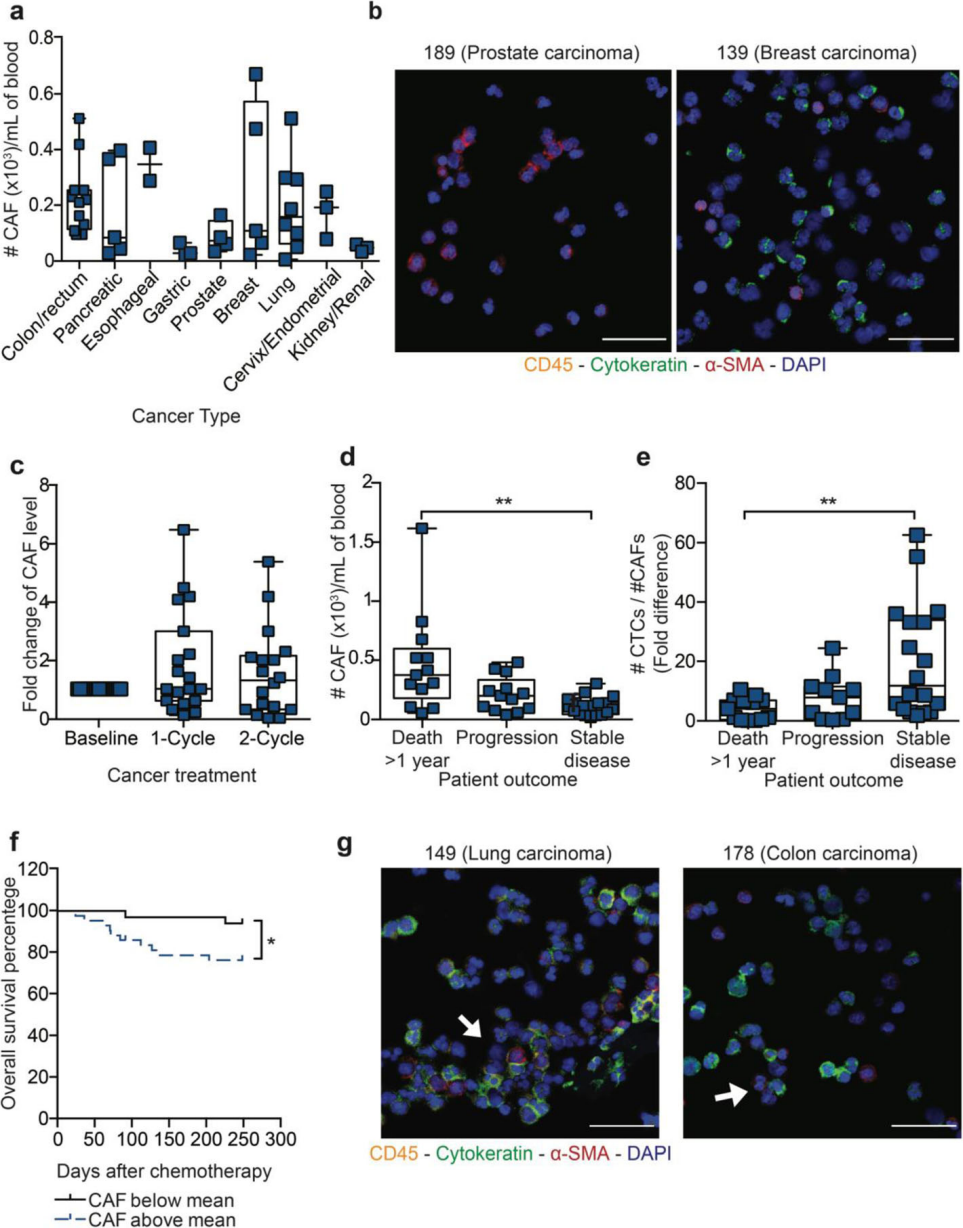
Fluctuation of CAF level in Met-pa receiving chemotherapy treatment. **a** Scatter dot plot represents baseline CAF counts found in blood samples from Met-pa across a spectrum of cancer types (median ± SD, *N* = 44 from 45 patients). **b** Immunofluorescence photomicrographs of CAFs isolated from blood samples (CD45 is yellow, α-SMA is red, cytokeratin is green and DAPI is blue). Scale bar is 40 μm. **c** Box and whisker charts show the fold change in CAF counts after the patients received 1 and 2 cycles of chemotherapy (median ± range, *N* = 58 from 23 patients). No significant increase of CAF counts (*P* < 0.7436) after chemotherapy treatment was determined using a Friedman test. **d** Box and whisker plots represents CAF counts with respect to the clinical outcome of Met-pa (median ± range, *N* = 44 from 44 patients). Significance of CAF level (**P = 0.0017) in the cancer prognosis was calculated using a Kruskal-Wallis test. **e** Box and whisker plots represent the fold change of CTC/CAF counts in the Met-pa (median ± range, *N =* 44 from 44 patients). Significance of CTC:CAF ratio (**P = 0.0057) in the cancer prognosis was calculated using a one-way ANOVA test. **f** Survival curve represents the overall survival percentage of Met-pa based on the CAF count at baseline using the mean value for CAF counts (*N* = 44 from 44 patients). Significant effect of CAF counts (*P = 0.0223) in predicting the survival probability for Met-pa was determined using a Log-rank (Mantel-Cox) test. **g** Immunofluorescence photomicrographs of CAFs incorporated in CTC aggregates (CD45 is yellow, α-SMA is red, cytokeratin is green and DAPI is blue). Scale bar is 40 μm

To investigate the importance of circulating CAF levels in Met-pa during cancer prognosis, blood samples were collected from 45 patients from which CAFs were isolated and enumerated using the following criteria: (i) negative for CD45, (ii) positive for α-Smooth Muscle Actin (α-SMA) and (iii) intact for nuclear staining via DAPI. Blood from healthy donors was used as a negative control where no CAFs were found (Additional file 1A). Of 45 patient samples, 98% (44 of 45) contained over 17 CAF/mL of blood (Additional file 1B and C). We observed a variation in CAF levels with cancer type, where renal and gastric cancer showed lower CAF levels (< 60 CAF/mL) in contrast to breast, lung, prostate, and colorectal cancer which showed higher CAF levels (> 200 CAF/mL), however these comparisons do not, in general, reach statistical significance due to the number of samples analyzed of each cancer type (Fig. 2a and b). This variation in CAF count in different patients and cancer types may in fact arise from different mechanisms of CAF cell emergence. To determine the temporal fluctuation of CAF levels in Met-pa through chemotherapy treatment, we enumerated the CAF level in 26 of patients before, after 1 cycle and 2 cycles of chemotherapy. It was determined that CAF levels don’t fluctuate over chemotherapy treatment in this cohort of patients (Fig. 2c). During chemotherapy, only 12% (3 of 26) of patients showed a continuous disease progression via CT scan in which CAF levels increased gradually. At the end of the study, the clinical outcome information was collected and correlated with the initial CAF levels. Importantly, it was determined that increasing CAF level correlated with greater or more extensive cancer progression and lower probability of overall survival (Fig. 2d, e and f). This is expected due to the theory that tumor cells migrate in aggregate form with stromal cells such as CAFs, consistent with the aggregates we have observed in several Met-pa (Fig. 2g) [5, 13]. Collectively, the positive correlation between circulating CAFs and poorer clinical outcome and lower survival in Met-pa was observed in a variety of cancer types diagnosed at metastatic stage. Furthermore, it was found that chemotherapy didn’t affect the level of CAF in the circulation of Met-pa. The next question to address was whether CTC levels showed a similar correlation with cancer prognosis, overall survival and chemotherapy treatment in this cohort of patients.

### No significant correlation of CTC level with cancer prognosis in Met-pa

**Fig. 3:**
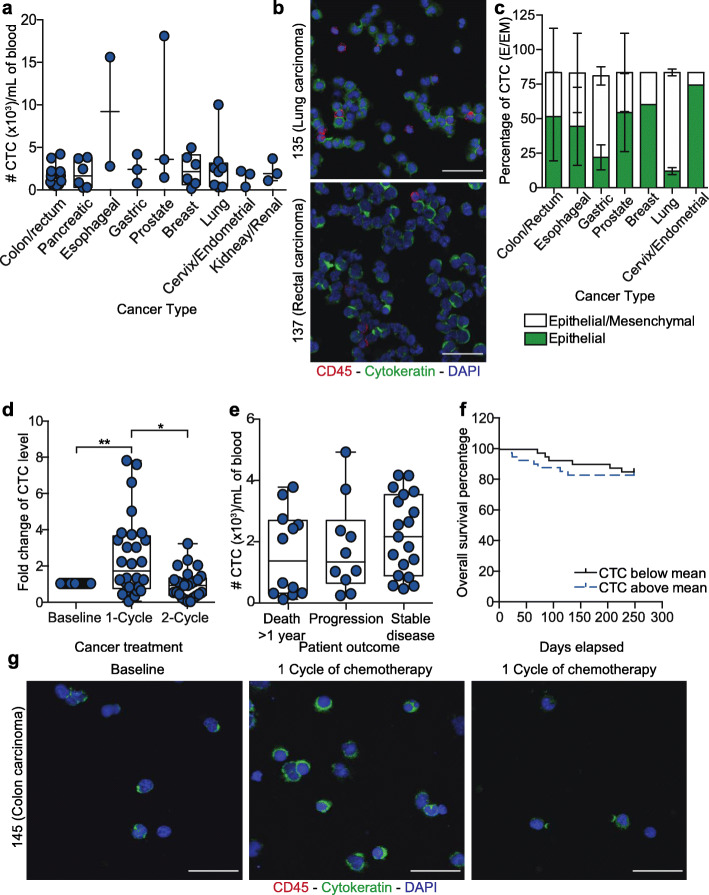
Fluctuation of CTC levels in Met-pa receiving chemotherapy. **a** Scatter dot plot represents the CTC levels in Met-pa with a spectrum of cancer types (median ± range, *N* = 48 from 48 patients). **b** Immunofluorescent photomicrograph of CTCs isolated from 2 patients with metastatic rectal and lung cancer (CD45 is red, cytokeratin is green and DAPI is blue). Scale bar is 40 μm. **c** Stack column charts represent the percentage of CTCs displaying epithelial (E) and both (E/M) phenotypes across cancer type (mean, *N* = 24 from 12 patients). **d** Box and whisker charts display the fold change in CTC levels after chemotherapy (median ± range, *N* = 83 from 30 patients). Significance increase of CTC counts (**P = 0.0047 and *P = 0.0103) after chemotherapy was calculated using a Wilcoxon test. **e** Box and whisker plots display the CTC levels at baseline in patients with different outcomes (death 1–12 months, disease progression but alive at 12 months, stable disease at 12 months) (median ± range, N = 48 from 48 patients). CTC levels were not significantly different (*P* = 0.3143) between the death within 1–12 months and stable disease groups, as calculated with a Kruskal-Wallis test. **f** Survival curve displays the overall survival percentage of Met-pa based on the initial CTC level. The mean value of CTC counts was used for the survival curve (*N* = 48 from 48 patients). Non-significant effect of CTC counts (*P* = 0.4492) in predicting the survival probability for Met-pa was determined using a Log-rank (Mantel-Cox) test. **g** Immunofluorescence photomicrograph of CTCs isolated from a patient with metastatic colon cancer before, after 1 cycle and 2 cycles of antimetabolite-based chemotherapy (CD45 is red, cytokeratin is green and DAPI is blue). Scale bar is 40 μm

**Fig. 4:**
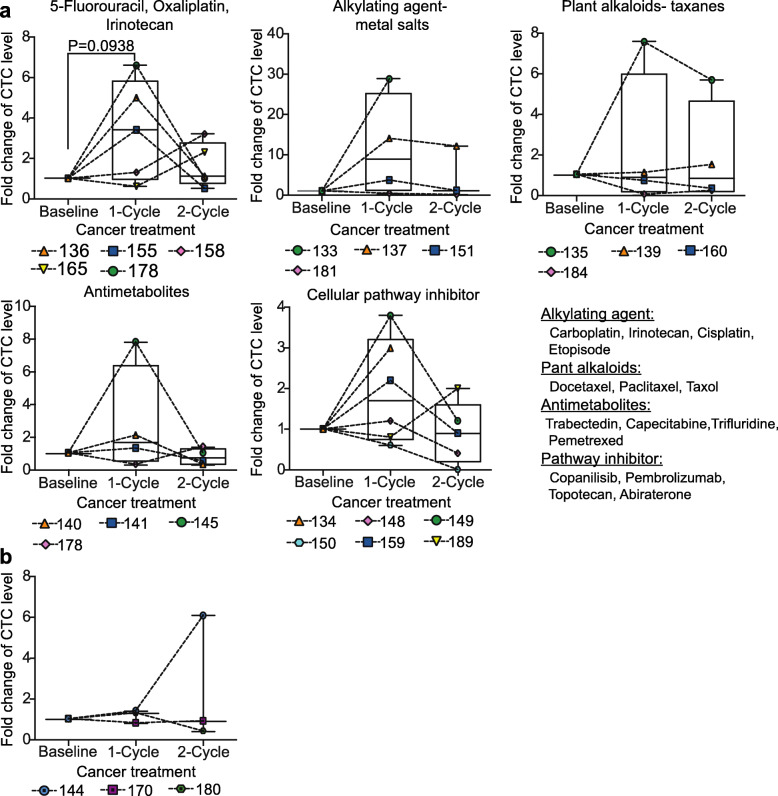
CTC mobilization with different chemotherapeutic regimens. **a** Box and whisker charts show the fold change in CTC counts after 1 and 2 cycles of treatment with: A combination of Fluorouracil, Oxaliplatin and Irinotecan, or alkylating agent-, plant alkaloids-, antimetabolite- and inhibitor-based chemotherapy. Lines connect individual patients (median ± range, *N* = 72 from 30 Met-pa). The changes in the median CTC level over the course of each treatment was tested for significance with a paired Wilcoxon test. **b** Box and whisker plots represent the fold change in CTC counts post-chemotherapy in patients showing continuous progression of cancer. Lines connect individual patients (median ± range, *N* = 9 from 3 Met-pa). The change in median CTC level post-treatment was not significant using a paired Wilcoxon test (0.500 < *P* > 0.999)

To investigate the correlation of CTC levels with cancer prognosis in Met-pa, blood samples were collected from 48 patients. CTCs were isolated from patient blood and enumerated using the following criteria: (i) negative for CD45, (ii) positive for cytokeratin and (iii) intact nuclear staining via DAPI. Blood from healthy donors was used as a negative control and no CTCs were detected in these samples (Additional file 2A). It was determined that 100% of patient samples contained over 94 CTCs/mL of blood (Additional file 2B and C). CTC levels varied between cancer type; lower CTC counts were found in patients diagnosed with pancreatic, cervical and endometrial cancer. However, higher CTC counts were found in patients diagnosed with metastatic prostate, lung and esophageal cancer (Fig. 3a). CTCs were observed as both single CTCs and CTC clusters (Fig. 3b). The isolated CTCs were composed of heterogeneous CTC populations displaying epithelial and mesenchymal (EM) plasticity. This was determined by immunostaining for common EM biomarkers including cytokeratin (epithelial marker) and vimentin (mesenchymal marker). In cervical, endometrial, colorectal, breast, prostate and esophageal cancer, a least 40% of the CTCs exhibited both epithelial and mesenchymal markers. However, over 70% of gastric and lung cancer displayed over 70% of CTCs with a mixed epithelial/mesenchymal phenotype (Fig. 3c). It was found that after Met-pa underwent chemotherapy, CTC level increased over twofold compared to the CTC level at baseline in 87% (26 of 30) of patients. After 2 cycles of chemotherapy, the CTC levels normalized in 63% (19 of 30) of patients (Fig. 3d and g). When the cohort of patients was grouped by chemotherapy, patients receiving a combination of 5-Fluorouracil, Irinotecan and Oxaliplatin showed a slight increase in CTC levels (around 3-fold) after 1 cycle of chemotherapy compared to its level at baseline. However, this effect was not observed in patients receiving other chemotherapy treatments (taxanes, plant alkaloids, antimetabolites or cellular pathway inhibitor) (Fig. 4a). Regarding patients that exhibited ongoing cancer progression, the CTC level slightly increased throughout chemotherapy treatment (Fig. 4b). At the end of treatment, based on the clinical outcome information, CTC levels did not correlate with cancer prognosis and poor probability of overall survival in these Met-pa (Fig. 3e and f). In summary, we determined a strong correlation of chemotherapy treatment and increasing CTC levels post-treatment. Interestingly, CTC levels did not correlate with the cancer prognosis and worse clinical outcome. Due to the importance of the cell aggregates of CAFs and CTCs in the circulation of Met-pa in promoting cancer progression, it stands to reason that a CTC or CAF targeted therapy should be implemented along with chemotherapy treatment to eradicate the CTC /CAF increase observed. Thus, we investigated the efficacy of our previously developed TRAIL-based liposomal therapy as a CTC targeted approach to neutralize CTCs in the bloodstream.

### TRAIL-based liposomal therapy kills CTCs in metastatic cancer patient blood

**Fig. 5:**
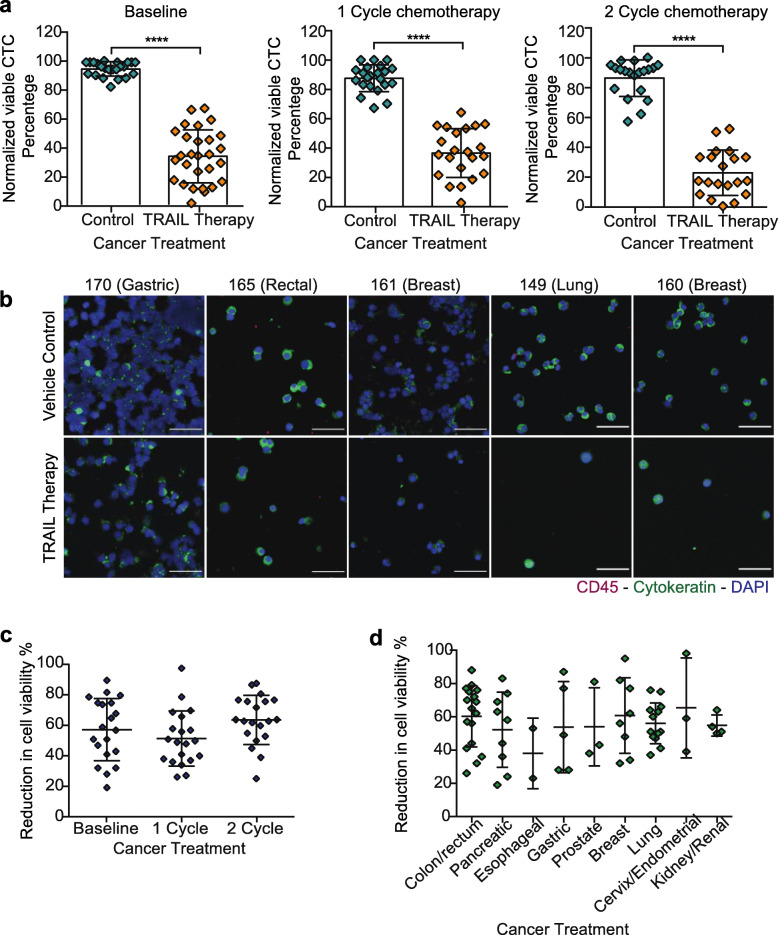
TRAIL-based liposomal therapy killed CTCs from metastatic cancer patients. **a** Scatter dot charts represent the normalized cell viability percentage of CTCs treated with vehicle control and TRAIL-based liposomal therapy in flowing blood for 4 h at different time points of cancer treatment: Baseline, 1 cycle of chemotherapy and 2 cycles of chemotherapy (mean ± SD, *N* = 68 from 26 cancer patients). Significant decrease (****P < 0.0001) of CTC in treated samples was calculated using a paired *t* test. **b** Immunofluorescence photomicrographs of CTCs remaining after being treated in flowing blood with vehicle control and TRAIL-liposomal therapy in patients with gastric, rectal, breast and lung cancer (CD45 is red, Cytokeratin is green and DAPI is blue). Scale bar is 40 μm. **c** Scatter dot charts represent the reduction in cell viability percentage in treated samples with TRAIL-based liposomal therapy before and after chemotherapy (mean ± SD, *N* = 65 from 26 patients). Non-significant increase *(P* = 0.1424) in killing rate of CTC after receiving 1 or 2 cycles of chemotherapy was calculated using one-way ANOVA (repeats matched) test. **d** Scatter dot plots represent the variation in cell viability reduction percentage in treated samples with TRAIL-liposomal therapy by cancer type (mean ± SD, *N* = 65 from 26 patients). There were no significant differences (*P* = 0.2514) in the mean CTC reduction by cancer type as determined with one-way ANOVA

**Fig. 6:**
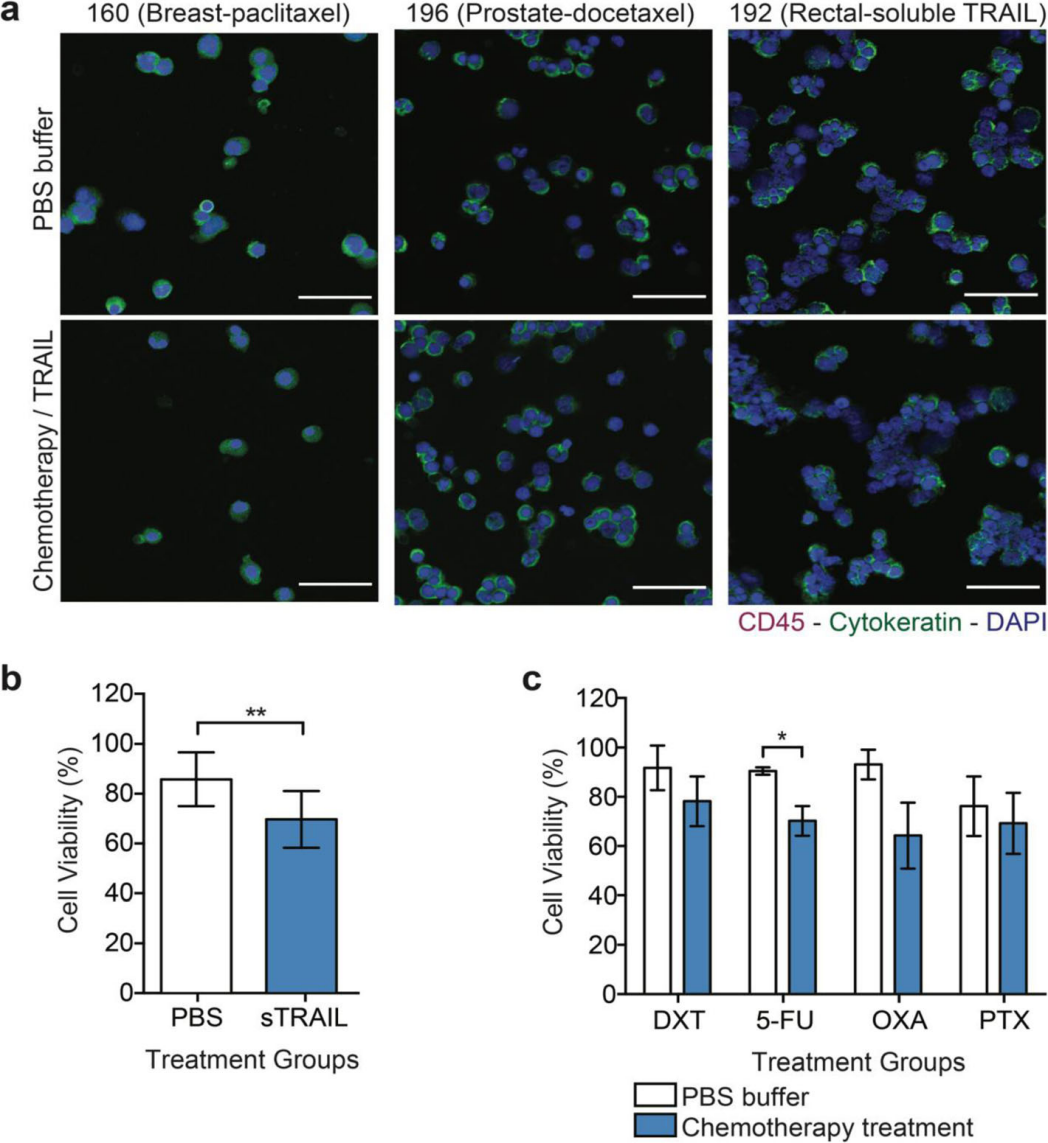
Minimal reduction of CTC viability by chemotherapy and soluble TRAIL. **a** Bar graphs display the viable CTC percentage in treated samples with PBS and chemotherapeutic agents (Docetaxel-DXT, 5-Fluorouracil-5-FU, Oxaliplatin-OXA, Paclitaxel-PTX) in Met-pa (mean ± SD, *N* = 24 from 10 patients at different time points of chemotherapy treatment). Significant reduction in (*P = 0.0317) in CTC viability due to the chemotherapy was determined using a paired *t* test. **b** Bar graphs represent the CTC viability percentage in samples treated with PBS and soluble TRAIL (mean ± SD, *N* = 12, from 6 patients). Significant reduction in CTC viability percentage (**P = 0.0048) by soluble TRAIL was calculated using a paired *t* test. **c** Immunofluorescence photomicrographs of viable cells obtained from breast, prostate and colorectal cancer patients after ex vivo treatment with chemotherapies, including docetaxel, paclitaxel, and soluble TRAIL (CD45 is red, cytokeratin is green and DAPI is blue). Scale bar is 40 μm

To determine the efficacy of the TRAIL delivery approach to kill CTCs, approximately 1 to 2 mL of 68 blood samples from 26 Met-pa were treated with 40 μL (290 μg/mL of TRAIL) of vehicle control and TRAIL-based liposomal therapy in a cone-and-plate viscometer for 4 h at different time points, including: Baseline, 1 cycle of chemotherapy and 2 cycle of chemotherapy. The CTCs were then isolated and viable CTCs enumerated using the following criteria: (i) negative for CD45, (ii) positive for cytokeratin, (iii) intact for nuclear staining via DAPI and (iv) negative for necrosis via staining with propidium iodide (PI). We found that TRAIL-liposomal therapy killed over 60% of CTCs in Met-pa with a range of cancer types. Importantly, despite the increase in CTC levels induced by chemotherapy, the TRAIL therapy consistently killed CTCs after patients underwent 2 cycles of chemotherapy (Fig. 5a and b). When the reduction in cell viability percentage through chemotherapy treatment was examined, the CTCs showed slightly similar sensitivity to TRAIL cytotoxicity compared to CTCs at baseline (Fig. 5c). Interestingly, it was found that the efficacy of TRAIL-liposomal therapy to kill CTCs in flowing blood fluctuated across cancer type. It was determined that the TRAIL therapy efficiently killed CTCs from colorectal, breast, cervix, endometrial and lung cancer. However, in esophageal cancer this therapy killed CTCs to a lesser degree (Fig. 5d). Blood samples from 5 Met-pa were treated with PBS and soluble TRAIL (290 μg/mL) to confirm that the efficacy of TRAIL-based liposomal therapy to target and kill CTCs in blood under shear conditions was enabled by the presence of E-selectin, which enhanced targeted TRAIL delivery. As expected, soluble TRAIL did not significantly decrease CTC viability. Soluble TRAIL killed approximately 16% of the CTC from Met-pa (Fig. 6a and b). Considering that Met-pa undergo chemotherapy as the first-line of treatment, we investigated the efficacy of chemotherapy to reduce CTC viability under the same experimental conditions. Blood from 9 Met-pa (collected at baseline, after 1 and 2 cycles of chemotherapy) with their respective chemotherapeutic regimens were treated, including docetaxel, paclitaxel, 5-fluorouracil and oxaliplatin using the peak plasma concentration. As expected, the chemotherapy drugs killed less than about 30% of CTC (Fig. 6a and c). This finding demonstrates that the significant reduction of CTC viability is due to the efficacy of E-selectin to deliver TRAIL to CTCs under shear conditions. Together, these findings indicate that TRAIL-based liposomal therapy is a potential therapy that could be used to efficiently target and eradicate CTCs to enhance overall survival in patients diagnosed with advanced metastatic cancer.

## Discussion

The first line of treatment for many advanced-stage cancers is chemotherapy. Chemotherapy reduces disease symptoms and extends patient life expectancy [26]. In this study, we demonstrated that chemotherapy may induce CTC mobilization, as a single and cell aggregate, from the primary tumor and metastases into the bloodstream in Met-pa. After 2 cycles of chemotherapy, CTC levels normalized to baseline in most patients. It is likely that the dynamic change in CTC counts in the blood samples were mostly a result of the systemic response to the chemotherapy rather than disease progression, since the Met-pa showed stable disease with no progression at each time point. With respect to the CAF levels, these did not fluctuate across chemotherapy treatment. In this study, only 13% of the patients showed no benefit from the chemotherapy treatment due to an ongoing cancer progression. For this small cohort of patients, the CTC and CAF levels gradually increased despite treatment.

Increasing CTC levels have been widely used as a clinical biomarker for disease progression and prognosis in metastatic cancer [27–29]. However, few studies have determined the correlation of CAF levels with disease progression in patients diagnosed with breast, prostate and colorectal cancer [11]. For the first time, we determined the presence of circulating CAFs in patients diagnosed with metastatic pancreatic, esophageal, gastric and renal cancer. Importantly, in this cohort of patients, the CAF levels showed a positive correlation with the worse probability of overall survival. In this cohort of patients, CAF were found as single circulating cells as well as interacting with CTCs in cluster form. These findings led us to hypothesize that CTC-CAF clusters, termed as collective migration units, could be a main precursor cancer progression. Several studies have shown that CAFs enhance the formation of metastases in distant organs by maintaining the epithelial phenotype in tumor cells, which is crucial to growth at distant sites [30, 31]. We found that CAF has a significant presence in the circulation but further studies should be done to determine the cellular mechanisms governing their fate. Based on the correlation of initial CAF level with cancer prognosis, we validated the potential use of this circulating cell level as a biomarker to predict cancer prognosis in Met-pa in the clinical setting.

Despite the increased CTC levels observed post-chemotherapy in Met-pa, we propose the use of CTC-based liposomal therapy as a promising adjuvant therapy to reduce the viability of CTCs in the circulation to prevent disease progression and enhance disease-free survival. Here we evaluated the efficacy of our previous TRAIL-liposomal therapy and determined that this therapy killed over 60% of CTCs from Met-pa. After patients received 2 cycles of chemotherapy, the TRAIL-therapy killed over 80% of CTC, which may indicate that chemotherapy sensitizes the tumor cells to TRAIL cytotoxic effect as previous studies have shown [32–34]. This observation led us to conclude that the potential use of this TRAIL therapy in combination with chemotherapy can eradicate the majority of CTCs; this reduction could extend the probability of survival in Met-pa [19].

## Conclusion

In conclusion, we demonstrate the CTC mobilization induced by chemotherapy in Met-pa. This CTC mobilization could occur as cell aggregates, which may have a higher metastatic potential than single CTCs. Importantly, we found a correlation between CAF levels, worse cancer prognosis and poor probability of overall survival, which indicates the potential use as a predictor biomarker in the clinical setting. Eradication of CTCs with TRAIL-based liposomal therapy supports its potential to reduce metastatic burden and ultimately increase the life expectancy in patients diagnosed with cancer at late stage.

## Supplementary information

**Additional file 1.** Blood samples were collected from healthy donors where no CAFs were found.

**Additional file 2.** Blood samples were collected from healthy donors where no CTCs were found.

## Data Availability

The datasets used and analyzed in this study is available from the corresponding author by reasonable request.

## Abbreviations

Met-pa: Metastatic cancer patients
CTCs: Circulating tumor cells
CAFs: Cancer-associated fibroblast
TRAIL: TNF-related apoptosis inducing ligand
