# Supplementary material for: Chemotherapy-induced release of circulating-tumor cells into the bloodstream in collective migration units with cancer-associated fibroblasts in metastatic cancer patients
Source: BMC Cancer. 2020 Sep 11;20:873. doi: 10.1186/s12885-020-07376-1 (PMC7488506; doi:10.1186/s12885-020-07376-1)
Supplement: Supplementary file 1 — Additional file 1. Blood samples were collected from healthy donors where no CAFs were found. [file 12885_2020_7376_MOESM1_ESM.docx]

**Additional Files:**


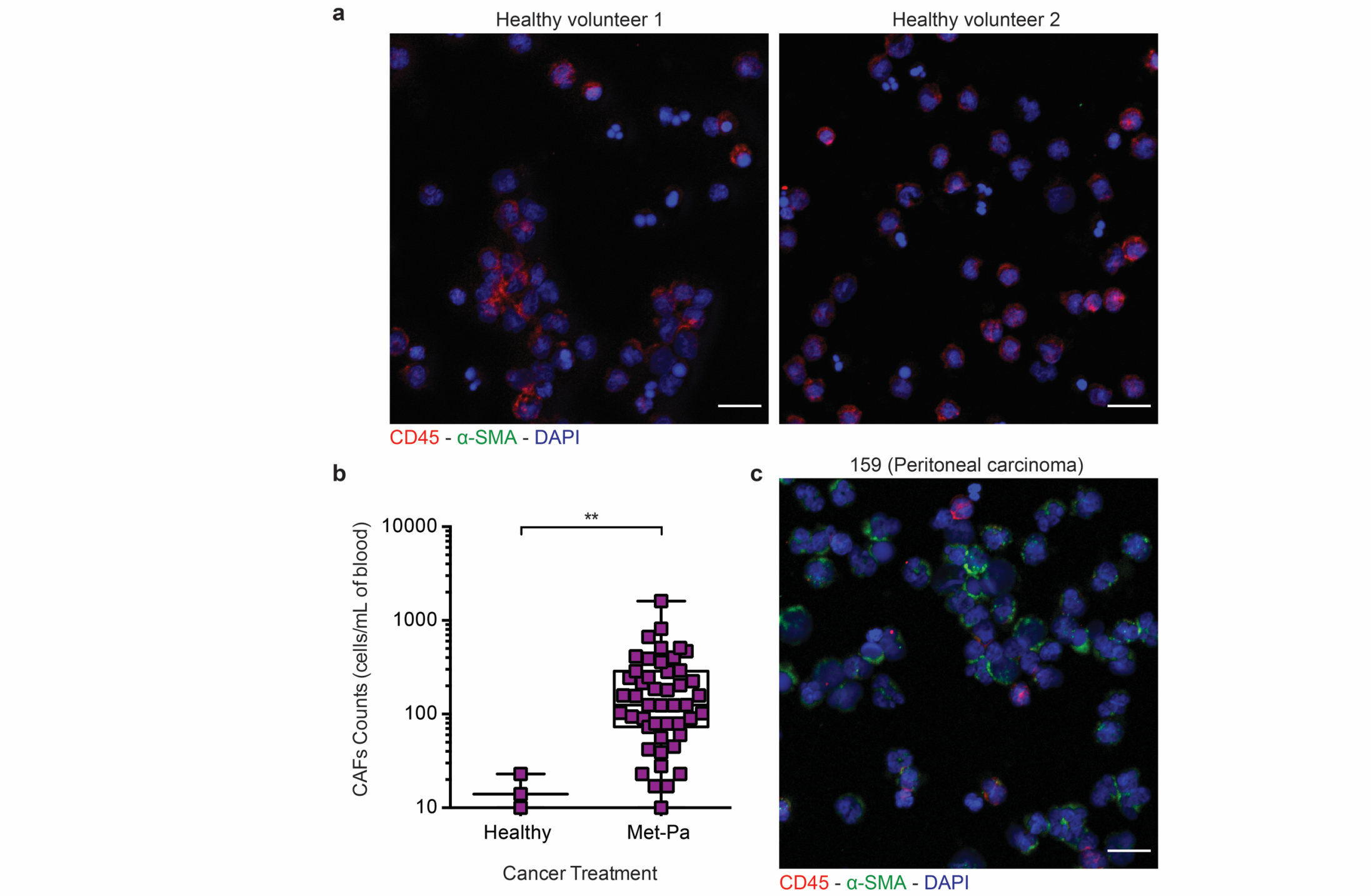


**Additional file 1: Blood samples were collected from healthy donors where no CAFs were found.** **(A)** Immunofluorescent staining of CAFs from blood collected from healthy volunteers and processed using the same experimental procedures as the patients’ samples were handled (red is CD45, green is α-SMA and blue is DAPI). Scale bar is 20 µm. **(B)** Box and whisker plot represents the CAF counts in healthy donors and Met-Pa from a spectrum of cancer types (median ± range, *N=*47 from 44 Met-pa and 3 healthy donors). Significant level of CAFs found in Met-pa (***P=*0.0018) compared with healthy donors was determined using a Mann-Whitney test. **(C)** Immunofluorescent staining of CAFs from peritoneal carcinoma patient’s blood sample (red is CD45, green is α-SMA and blue is DAPI). Scale bar is 20 µm.
