# Supplementary material for: Chemotherapy-induced release of circulating-tumor cells into the bloodstream in collective migration units with cancer-associated fibroblasts in metastatic cancer patients
Source: BMC Cancer. 2020 Sep 11;20:873. doi: 10.1186/s12885-020-07376-1 (PMC7488506; doi:10.1186/s12885-020-07376-1)
Supplement: Supplementary file 2 — Additional file 2. Blood samples were collected from healthy donors where no CTCs were found. [file 12885_2020_7376_MOESM2_ESM.docx]

**
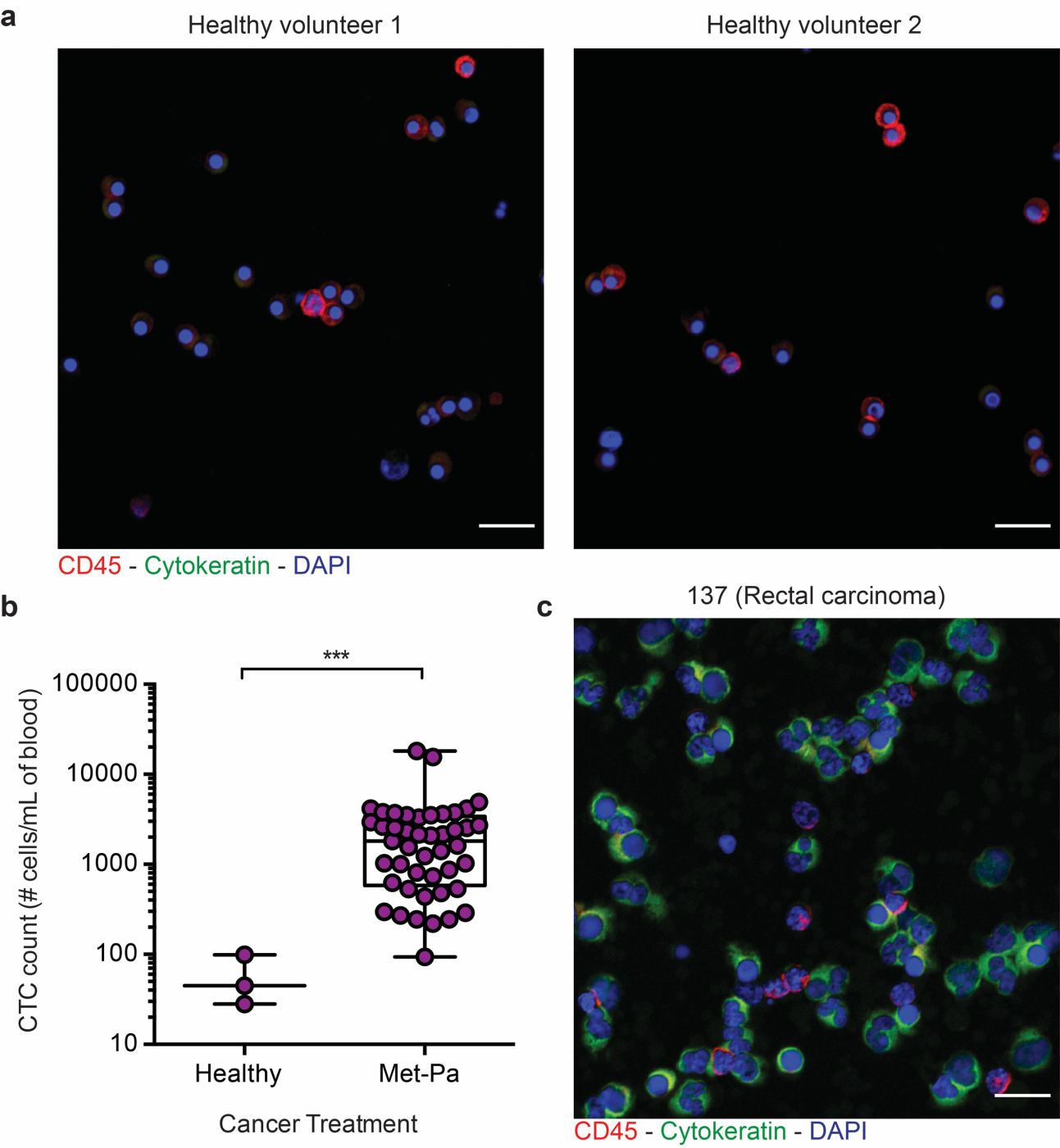
**

**Additional file 2: Blood samples were collected from healthy donors where no CTCs were found.** **(A)** Immunofluorescent staining of CTCs from blood collected from healthy volunteers and processed using the same experimental procedures as the patients’ samples were handled (red is CD45, green is Cytokeratin and blue is DAPI). Scale bar is 20 µm. **(B)** Box and whisker plot represents the CTC counts in healthy donors and Met-Pa from a spectrum of cancer types (median ± range, *N=*50 from 47 Met-pa and 3 healthy donors). Significant level of CTCs in Met-pa (****P=*0.0002) compared to healthy donors were calculated using a Mann-Whitney test. **(C)** Immunofluorescent staining of CTCs from rectal carcinoma patient’s blood sample (red is CD45, green is cytokeratin and blue is DAPI). Scale bar is 20 µm.
